# An open-source linear actuated-quartz tube furnace with programmable ceramic heater movement for laboratory-scale studies of combustion and emission

**DOI:** 10.1016/j.ohx.2026.e00790

**Published:** 2026-05-11

**Authors:** Casey Coffland, Ryan Bixler, Elliott Gall

**Affiliations:** Department of Mechanical and Materials Engineering, Portland State University, 1930 SW 4^th^ Avenue, Portland, OR 97201, USA

**Keywords:** Smoldering, Flaming, Combustion emission research, Biomass burning, Wildland-urban interface fires, Air quality

## Abstract

The Linear Actuated Quartz Tube Furnace (LA-QTF) is an instrument engineered to heat and combust materials under controlled conditions, capable of achieving flaming and smoldering states. A ceramic ring furnace is linearly actuated parallel to the length of a quartz tube. The mode of combustion depends on temperature, fuel composition, and oxygen availability; the LA-QTF regulates combustion by controlling the temperature and position of the ring furnace, and airflow within the tube. The LA-QTF can maintain temperatures between 23 °C and 530 °C for extended time periods, with stable temperatures over long-duration (∼120 min) experiments. Flow rate is dependent on the chosen pump; we designed for an upper limit of 3.5 LPM, though the tube furnace can accommodate lower or higher flows. To quantify CO_2_ emissions from combustion processes, the design incorporates lower-cost CO_2_ sensors at the inlet and outlet of the quartz tube. Real-time furnace temperature and CO_2_ data are printed to the Arduino serial monitor, providing users with live feedback and data logging capabilities. We aim to enable users to safely and consistently heat and combust different materials under smoldering and flaming conditions. All design and software files are open source, facilitating replication and customization to suit users’ specific needs.

## Highlights

• Open-source linear actuated quartz tube furnace.
• Translating ring furnace controls heating dynamics.
• Achieves flaming and smoldering combustion states.
• Lab studies of air pollution from wildfire and WUI fire.
• Open-source hardware built for around $3,000.

## Graphical abstract

**Figure:**
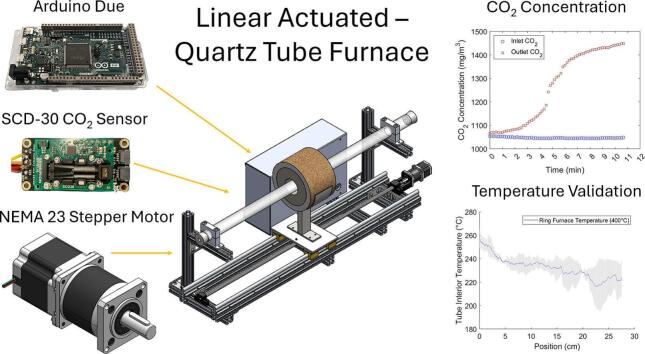

Specifications table.

Table 1.

**Table 1:** Specifications table for the Linear Actuated Quartz Tube Furnace (LA-QTF).

Hardware name	Linear Actuated Quartz Tube Furnace (LA-QTF)
Subject area	Engineering and materials science
Hardware type	Mechanical engineering and materials science
Closest commercial analog	No commercial analog is available
Open source license	CC-By Attribution 4.0 International
Cost of hardware	∼$3,000
Source file repository	https://doi.org/10.17605/OSF.IO/452PN

## 1 Hardware in context

**Fig. 1:**
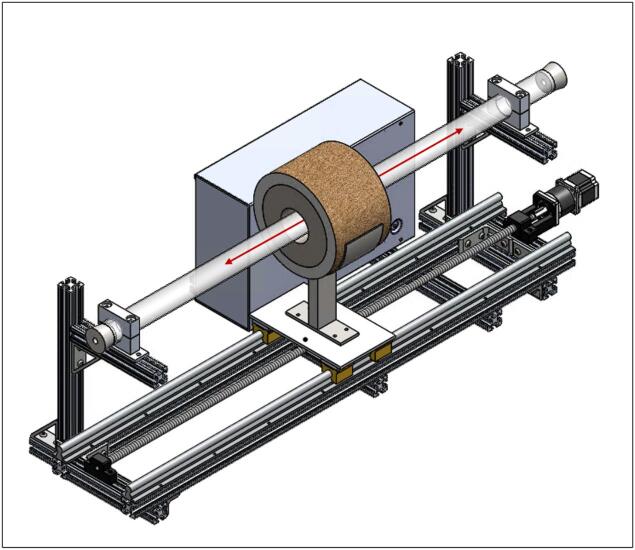
Linear Actuated Quartz Tube Furnace (LA-QTF) assembly.

The Linear Actuated Quartz Tube Furnace (LA-QTF) designed here enables control over heating and combustion of materials placed within the quartz tube. The central feature of the LA-QTF is a linearly actuated ring furnace capable of translating over the length of a quartz tube, as shown in Fig. 1. The position or velocity of the ring furnace is actuated via a NEMA 23 stepper motor, which creates local heating of a section of the tube furnace as it traverses the length of the tube. Since the stepper motor is connected to a 100:1 gearbox for slow and precise movement, the LA-QTF is capable of heating or combusting material placed in the tube furnace over the duration of the movement; this allows for generation of heat and emissions over periods ranging from ∼ 20 min to several hours, variable depending on the chosen gearbox ratio. Because the furnace traverses slowly over the tube, the LA-QTF is capable of consistent heating/combusting of a fraction of the test material; this scale of heating/combustion enables studies of emissions and heating appropriate for laboratory settings where large-scale combustion may be infeasible. The inlet of the quartz tube is connected to a gas-phase pump. The flow rate of the pump and the temperature of the ring furnace are both controllable to modulate the air supplied to the heating/combustion process; modifying these parameters creates specific combustion conditions including smoldering and flaming modes. For information on intended operating environment, power requirements, and specifications of the LA-QTF, see Table 2.

**Table 2:** Intended operating environment, power requirements, maximum furnace translation speed, and maximum operating temperature of the Linear Actuated Quartz Tube Furnace (LA-QTF).

Intended operating environment	Laboratory setting at 1 atm, 20–30 ℃, 20–50% RH
Maximum furnace translation speed	1.2 $\frac{\mathrm{cm}}{\min}$
Maximum furnace temperature	700 ℃
Power requirement	120 V, 20 A, AC outlet

Combustion and air quality research often aim to characterize a heating or combustion process quantitatively, so that conditions may be compared across experiments or a lab-scale process scaled to a real-world process. One important parameter for characterizing a combustion process is modified combustion efficiency (MCE), which is a key indicator of the combustion mode. The LA-QTF has CO_2_ sensing capabilities via two low-cost sensors (SCD-30, Sensirion), but due to the unreliability of low-cost CO sensors, an external CO monitor is required to calculate MCE. While MCE itself is not a feature of the LA-QTF, consistent MCE values show that this instrument can reproduce combustion conditions reliably. MCE can be characterized in the LA-QTF using the included CO_2_ sensors and an external CO monitor via Eq. (1):

(1) $$\mathrm{MCE}=\frac{E_{co_{2}}}{E_{co_{2}}+EE_{\mathrm{co}}}\times 100$$

Where MCE is the modified combustion efficiency (%), *E_CO2_* is the emission rate of carbon dioxide (CO_2_) (mg/h), *E_CO_* is the emission rate of carbon monoxide (CO) (mg/h), and 100 is a conversion factor from fraction to percentage.

Understanding material combustion chemistry and its impacts is important in many fields of research and practice. For example, controlled combustion and heating are necessary in fields such as combustion chemistry, environmental sciences, or toxicology [1], [2], [3], [4]. As humans are increasingly exposed to fires and smoke [5], there is a need for understanding how biomass and other burned and combusted materials emit pollution as a function of conditions like the MCE, since combustion emissions vary widely [6], [7], [8], [9]. The LA-QTF developed here allows for studies that explore heating and combustion of materials under specific modes, such as reproducing a wildland-urban interface (WUI) fire. The emissions from the combustion process can be collected for offline analysis or online via time-series data from external sensing devices. By characterizing the combustion emissions from different materials under specific modes of combustion, predictions of combustion products generated in the field can be made.

The LA-QTF design was based on a lab instrument used in a paper published by the U.S. EPA [10], in which combustion products from different materials were characterized using CO, CO_2_, and particulate matter sensors while also collecting samples for offline analysis in a multistage cryotrap. A similar experimental setup is shown in Blomqvist et al. [11], where rather than the ring furnace moving, the mass itself is moved through the furnace. Traditional materials science tube furnaces were used for combustion emission research where neither the mass nor the furnace translated [12], [13]. However, these prior studies only provided a simple diagram of their instruments in the manuscript—they did not provide detailed build instructions. The closest commercially available analog to the LA-QTF is the OTF 1200-X from MTI Corporation. A comparison between the LA-QTF and OTF 1200-X can be seen in Table 3. The OTF 1200-X uniformly heats the entire length of the tube and is generally intended for materials science applications where uniform heating is paramount. The OTF 1200-X does not have the ability to linearly translate the furnace along the length of the quartz tube. The LA-QTF addresses this limitation by using a shortened ring furnace, relative to static commercial quartz tube furnaces, and by allowing for control of the position or velocity of the ring furnace. Most natural combustion processes do not happen uniformly. An example of this is a wildfire: the leading edge of the wildfire propagates over the fuel source; these fire dynamics are amenable to a ring furnace where the heater translates over the quartz tube. Additionally, by being able to control the position or velocity of the ring furnace, the LA-QTF is capable of long-term smoke generation at a scale appropriate for laboratory settings.

**Table 3:** Comparison between the Linear Actuated Quartz Tube Furnace (LA-QTF) and the closest commercially available tube furnace, the MTI Corporation’s OTF 1200-X.

**Design**	**Cost ($)**	**Maximum Furnace** **Temperature (°C)**	**Tube Length (mm)**	**Translating Furnace**	**Open** **Source**	**CO_2_ Measurement**
LA-QTF	3,000	700	1200	Yes	Yes	Yes
OTF 1200-X	3500–4000	1200	450–600	No	No	No

The open-source design of our LA-QTF facilitates modification for inclusion of monitoring capabilities that are customizable and can be adapted to the experimental aims of the user. For example, in this report, we aim to help enable the determination of the MCE. For this, low-cost SCD-30 CO_2_ sensors are placed in custom housings at the inlet and outlet of the quartz tube. This CO_2_ sensor system measures the change in CO_2_ concentration due to the combustion process, enabling calculation of the CO_2_ emission rate. Ideally, precise CO sensors would be placed at the inlet and outlet of the quartz tube, enabling on-board and real-time calculation of MCE. To date, we have not identified a viable lower-cost CO sensor that could be outfitted similarly to the CO_2_ sensors used here. This is due, in part, to CO concentrations that are typically orders of magnitude lower than CO_2_ concentrations. In our experiments, CO time-series data is collected after the combustion emissions are diluted in an external chamber using a reference-grade CO monitor (Serinus 30, Ecotech); these experiments are not further described here. However, as noted previously, as sensing capabilities improve and change, the LA-QTF is designed to be retrofitted and modified by the user.

The LA-QTF presented here offers novel laboratory capabilities that address various limitations in systems available commercially and/or described in the research literature. Review of specifications and/or descriptions of these instruments shows one or more of the following limitations: 1) stationary ring furnace (not allowing for temperature gradients that enable long-term combustion/emissions experiments), 2) lack of support for combustion product transport, 3) not open source (no support for customizability in features or controls), 4) cost between $3,500 and $15,000, and 5) little to no build instructions or supporting documentation. The open-source LA-QTF addresses all these shortcomings while having material costs of ∼$3,000.

## 2 Hardware description

The LA-QTF furnace is a 15.2 cm (6 in) ring furnace that heats to 700 °C. The ring furnace is controlled by a PID controller to provide steady temperatures for extended periods of time (∼120 min) alongside a warm-up period (∼30 min to 650 °C). The ring furnace is mounted to a base connected to a linear actuator allowing the ring furnace to translate over the length of the quartz tube. The ring furnace temperature will be higher than the combustion temperature due to heat losses. This relationship was characterized and validated in Section 7.1.

The LA-QTF is equipped with a gas-phase pump to both provide combustion/heating air (or other gas-phase fluid as required) and transport the combustion products. In our case, the pump is pushing ambient air into the quartz tube, but this could be any gas the user has available. For example, the oxygen fraction could be varied through the introduction of a mixture of air or O_2_ with N_2_, to observe different combustion modes like varying MCE or pyrolysis.

The frame of the LA-QTF is made of 1515 extruded aluminum bars (80/20 Inc.), enabling additional external apparatus to be mounted when required. This is how the optional SCD-30 CO_2_ sensors (see Section 5.7) are mounted at the inlet and outlet of the quartz tube. Additionally, if the length of the quartz tube is not satisfactory, the LA-QTF can be shortened or lengthened to fit the needs of the user. Different-length aluminum bars and quartz tubes are commercially available from the vendors listed in the bill of materials.

The LA-QTF does not have an external enclosure around the quartz tube, allowing the user to physically witness the combustion process. Using the 80/20 mounting system stated above, the LA-QTF can be equipped with a camera to record the combustion process. For fire propagation research, this enables the user to review how the combustion process took place post-experiment. For commercial tube furnaces, the enclosure and heating component are opaque and span the length of the tube, preventing the user from seeing into it during the heating or combustion process.

The LA-QTF is equipped with programmable pins via the Arduino IDE. These pins can be utilized for sensors or to control the system. In the setup described, buttons are currently programmed to stop, start, and change the direction of the motor. Furthermore, a potentiometer is utilized to control the temperature of the ring furnace. If other needs are more important, the functions of the buttons or potentiometer can be reprogrammed. Additionally, these pins can be used for break-out sensors, similarly to how the SCD-30 CO_2_ sensor system has been described above.

Compared to commercial furnaces costing $3,500 or more, the LA-QTF can be built for under $3,000 using the materials described. The open-source LA-QTF provides precise control of temperature gradients, combustion product transport, mountability via 80/20, custom lengths to fit users’ needs, the ability to witness the combustion process, and programmable Arduino pins while being less expensive than commercially available tube furnaces. Additionally, if any parts were to fail on the LA-QTF, all parts are available online for replacement without the hassle of contacting proprietary companies for repair.

The LA-QTF has the following features and is relevant for the following applications:

● Safe small-scale controlled combustion conditions
● Characterization of material combustion emissions
● Programmable temperature setpoints for initiating heating, smoldering, or flaming combustion
● Combustion process visualization

## 3 Design files summary

The LA-QTF CAD files are CAD parts and an assembly meant to be referenced during construction of the LA-QTF. The SCD-30 CAD files consist mostly of custom parts that are meant to be 3D-printed. The Multiplexer_scd30 Arduino code is necessary to use the external SCD-30 system. The QTF_9-15–25 Arduino code is necessary to run the LA-QTF. The HardwareX Figures are meant to serve as a zoomable version of all figures if anything is unclear in the manuscript. The Bill of Materials serves as an organized Excel sheet to make obtaining and sorting the parts simple; it is also shown in Section 4. The Research Data Excel files back all claims made in the validation sections seen in Section 7.

## 4 Bill of materials summary

Table 5.

## 5 Build instructions

### 5.1 Build tips and safety

All SolidWorks assembly pack and go files are provided and can be found in Table 4. If any measurements are needed, please see the assembly files mentioned above. These files can be uploaded to Onshape if SolidWorks is not available. Onshape is free online CAD software that is available to the public. To see a better view of how each assembly is put together, parts can be hidden. Additionally, the list of all materials and their designators can be found in the Bill of Materials. In the instructions, the name of the part will be mentioned, then the corresponding designator will be in parentheses. This name and designator in parenthesis correspond to the name and designator in the Bill of Materials. If a part comes in a kit, the part will not have its own designator but will be called by the name of the part. Note that the only fasteners included are the 80/20 T-nuts and their matching bolts (1.10–11); this should cover most of the build, but others may be necessary depending on your application. Additionally, wire and wire shielding are not included in the Bill of Materials. The LA-QTF should be assembled in order that the build instruction sections are provided—that is, the frame should be assembled first. Also, it is recommended that adding the quartz tube to the assembly should be the last step to reduce risk of breakage when working on other components. The quartz tube is designed to be removed, so if a size fit is necessary, this can be done, but it should be removed before continuing assembly.

**Table 4:** Design files summary for the Linear Actuated Quartz Tube Furnace (LA-QTF).

**Design file name**	**File type**	**Open source license**	**Location of the file**
LA-QTF	CAD files	CC-By Attribution 4.0 International	https://osf.io/452pn/files/yknec?view_only = 29d59d5b2c374446bfb74a90c45bcfc0
SCD30	CAD files	CC-By Attribution 4.0 International	https://osf.io/452pn/files/k9xcf?view_only = 29d59d5b2c374446bfb74a90c45bcfc0
Multiplexer_scd30	Arduino code	CC-By Attribution 4.0 International	https://osf.io/452pn/files/q4rm5?view_only = 29d59d5b2c374446bfb74a90c45bcfc0
QTF_9-15–25	Arduino code	CC-By Attribution 4.0 International	https://osf.io/452pn/files/259w4?view_only = 29d59d5b2c374446bfb74a90c45bcfc0
HardwareX Figures	PowerPoint	CC-By Attribution 4.0 International	https://osf.io/452pn/files/q76kg?view_only = 29d59d5b2c374446bfb74a90c45bcfc0
Bill of Materials	Excel sheet	CC-By Attribution 4.0 International	https://osf.io/452pn/files/wehms?view_only = 29d59d5b2c374446bfb74a90c45bcfc0
Research Data	Excel sheets	CC-By Attribution 4.0 International	https://osf.io/452pn/files/hbnfv?view_only = 29d59d5b2c374446bfb74a90c45bcfc0

**Table 5:** Bill of Materials.

**Designator**	**Component**	**Number**	**Cost per unit (USD)**	**Total cost (USD)**	**Source of materials**	**Material type**	**Part #**
1.1	1515 Aluminum Extrusion − 48′’	2	41.75	83.5	80/20	Metal	NA
1.2	1515 Aluminum Extrusion − 6′’	2	7.73	15.46	80/20	Metal	NA
1.3	1515 Aluminum Extrusion − 20′’	2	19.07	38.14	80/20	Metal	NA
1.4	1515 Aluminum Extrusion − 14′’	2	14.21	28.42	80/20	Metal	NA
1.5	1515 Aluminum Extrusion − 16′’	2	15.83	31.66	80/20	Metal	NA
1.6	1515 Aluminum Extrusion − 9′’	2	10.16	20.32	80/20	Metal	NA
1.7	Tall Inside Corner	4	6.69	26.76	80/20	Metal	4301
1.8	Tall Gusseted Inside Corner	4	9.71	38.84	80/20	Metal	4336
1.9	Slotted Inside Corner	6	5.68	34.08	80/20	Metal	4295
1.10	Mounting Screws	50	0.47	23.5	80/20	Metal	3111
1.11	T-Nut	60	0.45	27	80/20	Metal	3278
1.12	Quartz Tube Base Mount	2	0.1	0.2	OnlineMetals	Metal	NA
1.13	Quartz Tube Lower Mount	2	0.67	1.34	OnlineMetals	Metal	NA
1.14	Quartz Tube Upper Mount	2	0.67	1.34	OnlineMetals	Metal	NA
2.1	16 mm Ball Screw	1	230.71	230.71	Mssoomm	Metal	SFU1604
2.2	16 mm Linear Rails	1	53.98	53.98	Creabygirls	Metal	SBR16/SBR16UU
2.3	NEMA 23 Stepper Motor and 100:1 Gear Ratio	1	91.84	91.84	Stepper Online	Other	23HS22-2804S-HG100
2.4	10 mm to 14 mm flexible shaft coupling	1	15	15	uxcell	Metal	a16121900ux039
2.5	NEMA 23 Ball Screw Mounting Bracket	1	53	53	CNCTOPBAOS	Metal	NA
2.6	Limit Switch Mount	2	0.5	1	Bambu Lab	Other	NA
2.7	Limit Switch	2	1	2	Amazon	Other	NA
2.8	Linear Actuator Stage	1	20	20	OnlineMetals	Metal	NA
2.9	Ball Screw Nut Mount	1	10	10	OnlineMetals	Metal	NA
2.10	Ball Screw Support	1	23.17	23.17	YedaHcy	Metal	EF12
2.11	Raft Spacers	4	1	4	OnlineMetals	Metal	NA
2.12	Ball Screw Connector	1	5	5	OnlineMetals	Metal	NA
3.1	Ring Furnace	1	599.8	599.8	Thermal Devices	Ceramic	TD-2297–2091
3.2	Ceramic Wool Insulation	1	159.3	159.3	Morgan Thermal Ceramics	Other	6224787004PLUS
3.3	Hold Down Spring	1	5.09	5.09	Prime Line	Metal	NA
3.4	Hold Down Chain	1	15.99	15.99	Koch	Metal	A14911
3.5	Heater Mount	1	20	20	OnlineMetals	Metal	NA
3.6	Heater Insulation Wrap	1	67.1	67.1	Techflex	Other	NA
3.7	Heater Box	1	3.81	3.81	Home Depot	Metal	5837112-25R
3.8	Thermocouple Mounting 1/4in Steel Rod	1	2	2	OnlineMetals	Metal	NA
4.1	Quartz Tube	1	99.7	99.7	Advalue Tech	Other	FQ-T-50–46-4
4.2	44–55 OD Tapered Silicone Plug	2	12.19	24.38	MECCANIXITY	Polymer	mef231229ee000160
4.3	Inlet/Outlet Tube	2	2.95	5.9	McMaster-Carr	Metal	4830 K114
5.1	Electronics Enclosure	1	115	115	Hoffman	Metal	ASE18X12X4
5.2	SSR Relay	1	133	133	Digi-Key	Other	RM1E23AA25
5.3	Temperature Controller	1	152	152	Autonics	Other	TK4S-T4CN
5.4	K-type Thermocouple	1	29.95	29.95	ScienceCompany	Other	NC-13979
5.5	Arduino Due	1	50	50	Arduino	Other	SAM3X8E ARM
5.6	Switching Power Supply 100 W 24 V 4.5A	1	20	20	Any Brand/Amazon	Other	NA
5.7	Digital Stepper Driver 1.0–4.2A 20-50VDC for NEMA 23	1	20	20	Stepper Online	Other	DM542T
5.8	RS-485 Signal Extender	1	9	9	JIUWU	Other	MAX485 RS-485
5.9	DK2.5 N Terminal Block	26	1.14	29.64	Dinkle	Other	DK2.5 N
5.10	SS2.5 N End Bracket	4	1.55	6.2	Dinkle	Other	SS2.5 N
5.11	DSS2.5-02P Jumper	9	1.01	9.09	Dinkle	Other	DSS2.5 N-02P
5.12	Din Rail, Slotted Aluminum-12″	1	9.99	9.99	International Connector	Metal	D357A11-305(2)
5.13	Power Converter, DC24V to DC5V, 3A, 15 W Step Down Reducer	1	15	15	EPBOWPT	Other	EA15-5 V
5.14	9ft, 14AWG, 15A/125 V AC Power Cable	1	15.99	15.99	Bergen Industries	Other	PS915143
5.15	12A 250 V Fast Blow Glass Fuse 6x30mm	1	4.99	4.99	Witonics	Other	NA
5.16	BK/HKP-HH Fuse Holder	1	10.29	10.29	Bussmann	Other	BK/HKP-HH
5.17	Push Buttons (set of 2)	1	12.98	12.98	TWTADE	Other	B07DYKX5TD
5.18	E-stop Button	1	10	10	KACON	Other	B30-81R
5.19	Control Station Box	1	12.49	12.49	UXCELL	Other	A18060400UX0 258
5.20	Flexible Conduit	1	25	25	Home Depot	Other	55,094,221
5.21	Conduit Fitting	2	4.16	8.32	Home Depot	Other	NA
5.22	30 mm 5 V Cooling Fan	2	2.8	5.6	Dorhea	Other	NA
5.23	Power Cord	1	20	20	Bergen Industries	Other	PS915143
5.24	Small Push Buttons (10 Pack)	1	8.25	8.25	Gikfun	Other	LYSB01E38OS7K-ELECTRNCS
5.25	Small Enclosure Top	1	1	1	Bambu Lab	Other	NA
5.26	Small Enclosure Bottom	1	1	1	Bambu Lab	Other	NA
5.27	Potentiometer	1	8	8	Flutesan	Other	NA
5.28	SSR Adapter Plate	1	15	15	Digi-Key	Metal	RHS00
6.1	SCD-30 (CO2 Sensor)	2	58.95	117.9	Adafruit	Other	4867
6.2	TCA9548A I2C Multiplexer	1	6.95	6.95	Adafruit	Other	2717
6.3	Airtight Chamber Mount	2	2	4	Bambu Lab	Other	NA
6.4	Airtight Chamber	2	10	20	NA	Other	NA
6.5	Arduino Uno	1	27.6	27.6	Arduino	Other	A000066
6.6	SCD-30 Mount	2	1	2	Bambu Lab	Other	NA

The quartz tube furnace features several points of concern to user safety that need to be considered during construction and operation. The furnace is powered by a 120 V AC electrical supply, which, if misused, poses shock and burn risk. Ensure all wiring, grounding, and connections are secure and correct prior to use. Regular inspection of the control box’s wiring should be performed to ensure no thermal damage has occurred from operation of the furnace. As several voltage levels are also present for the various components within the device, all power lines should be checked prior to being connected to prevent overvolting delicate components. Additionally, users should avoid contact with internal electrical components while the device is in operation to reduce the risk of shock. Any modifications made to the wiring should be performed with the device fully powered down.

The furnace itself reaches temperatures of up to 700 °C, representing a burn and fire hazard. Users must make sure the furnace sits atop a non-combustible and stable surface with adequate clearance from flammable or damageable materials/surfaces (>5 ft). The exterior surfaces of the furnace’s quartz tube can become extremely hot during operation and remain hot after shutdown. Proper personal protective equipment (PPE) in the form of heat-resistant gloves, glasses, and lab coats should be always worn when operating the device to mitigate these risks to users. We recommend the quartz tube furnace be placed behind a protective shield (e.g., acrylic shield) to prevent contact with the device and to mitigate risk of burns to users or occupants.

As the combustion of material fuel within the furnace generates smoke containing high concentrations of particulate matter and volatile organic compounds (VOCs), additional care must be taken to ensure users are shielded from the device’s combustion products, which are known to pose inhalation and exposure risks. All silicone fittings and tubing should be tightly sealed and snug with an inline pump, ensuring steady airflow through the device and into the target sealed chamber. The delivery of combustion products from the LA-QTF should be directed to an appropriate location, e.g., an air quality test chamber with controlled and known ventilation conditions. All combustible material should be placed and sealed within the quartz tube prior to heating. Experiments should be conducted within close range of exhaust ventilation to ensure capture of fugitive particulate and VOC emissions, which may result from loose seals or from the removal and reloading of combustion material. Ready access to a fire extinguisher and first aid equipment should be established prior to each operation, in case of emergency.

### 5.2 Frame

**Fig. 2:**
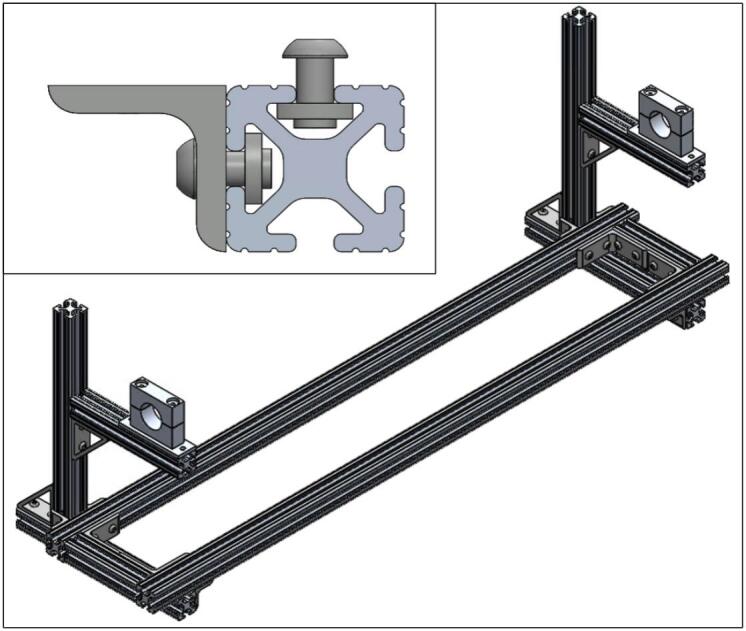
SolidWorks frame assembly (bottom right) and inset of T-nut fastened to 80/20 rail (top left).

All parts listed in the section, besides the quartz tube mounts (1.12–1.14), are available from 80/20. There are two pairs of custom aluminum quartz tube mounts (1.12–1.14). In our case, these mounts were fabricated out of aluminum using a mill; however, this could be manufactured commercially or 3D printed using suitable filament that can withstand high temperatures. For the frame assembly, all T-nuts and bolts should be sourced from 80/20 to ensure a sturdy frame. To see what the final frame assembly should look like, see the bottom right of Fig. 2. The length of the LA-QTF can be adjusted by altering the distance between where the quartz tube will sit on either side. The Bill of Materials lists a quartz tube that is 120 cm long; our temperature validation data covers 30 cm of the middle of the tube. The heating behavior will be consistent throughout the length of the tube. If altering the length is desired, it is recommended to move the left side (seen in Fig. 2) towards the center. The T-nuts (1.11) are fastened to the extruded 80/20 aluminum beams (1.1–1.6) by sliding the T-nut into the rail and threading the bolt in. As the bolt is rotated, the T-nut will try to rotate but will be caught by the inside of the 80/20 rail, preventing it from turning. This is how the T-nut can tighten against the rail of the aluminum beam. An example of what this should look like once the bolts are tightened can be seen in the inset image at the top left of Fig. 2.

**Fig. 3:**
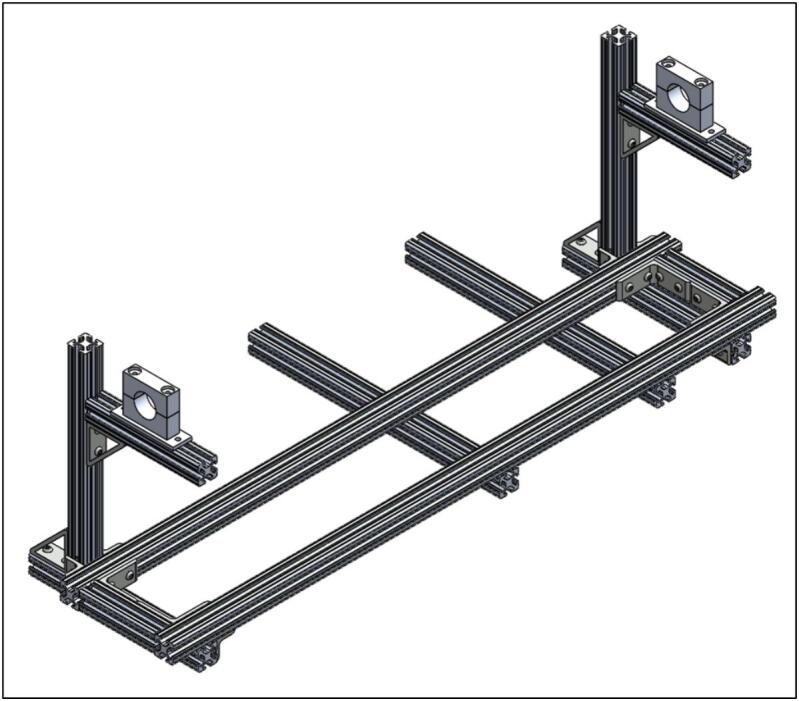
Frame assembly with electronics enclosure 80/20 attached.

If you wish to mount the electronics enclosure (5.1) to the quartz tube furnace itself, add the 80/20 20'' (50.8 cm) rails (1.3) to the bottom of the frame, as seen in Fig. 3. It is not required to have the electronics directly attached to the frame if another location is preferred.

### 5.3 Linear actuator

**Fig. 4:**
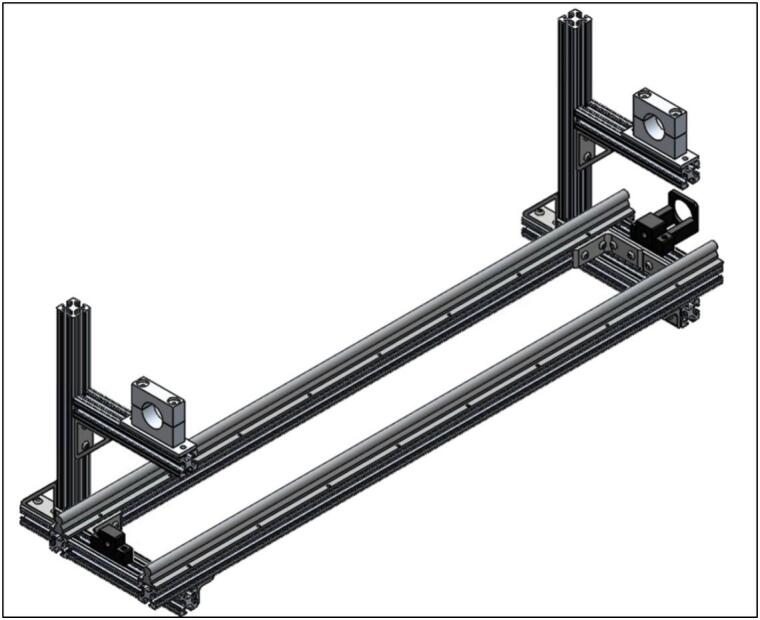
Ball screw mounting brackets and linear rails installed on frame.

To assemble the linear actuator, start by mounting the NEMA 23 ball screw mounting bracket (2.5) and the ball screw support (2.10) to the frame on the right- and left-side 80/20 cross bars (1.2), respectively. Be sure the provided ball bearing is installed into the ball screw support (2.10). Next, add the 16 mm linear rails (2.2) parallel to one another on the long sides of the frame. Holes will need to be drilled and tapped into the 48′’ (122 cm) 80/20 bars (1.1) to mount the rails. See Fig. 4 for the assembly at this stage.

**Fig. 5:**
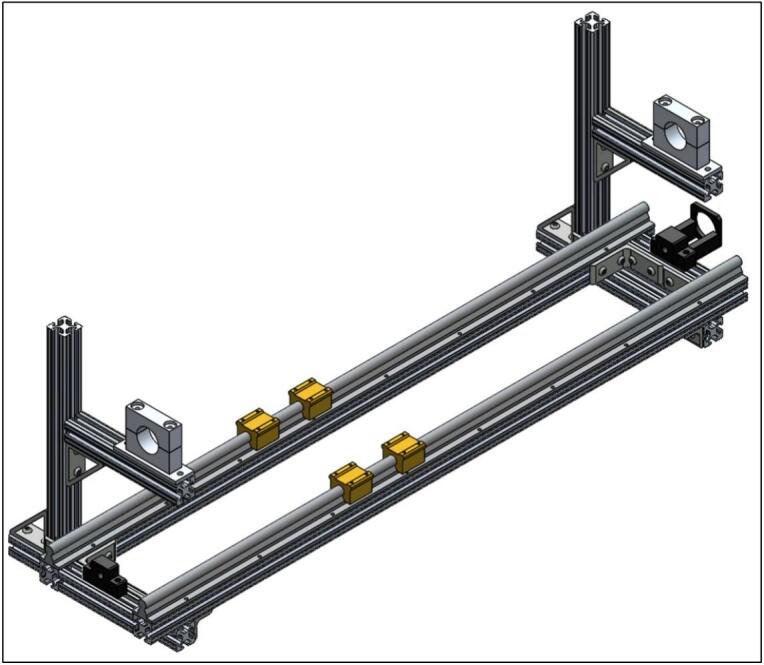
Heater slide rail mounts installed on linear rails.

Next, the heater slide rail mounts included with the linear rails (2.2) need to be slid onto the linear rails, as seen in Fig. 5.

**Fig. 6:**
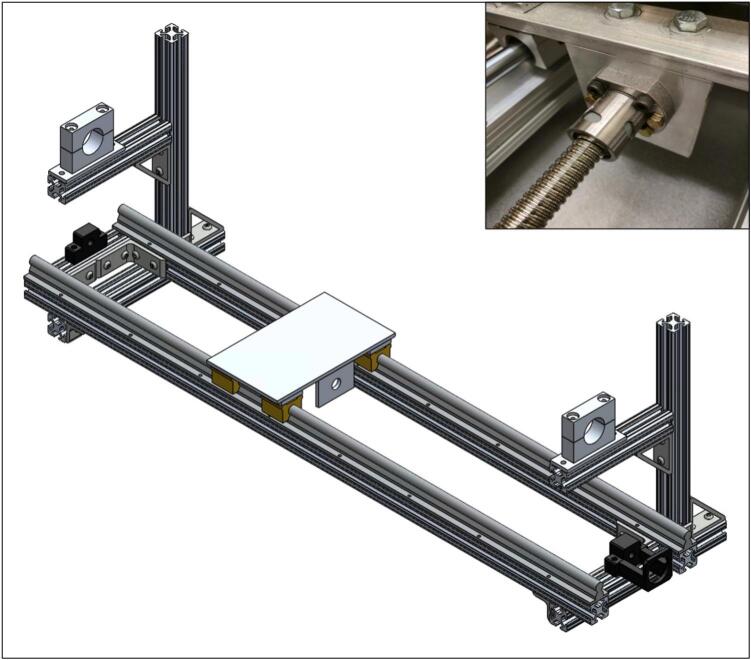
Linear actuator stage and ball screw connector mounted to heater slide rails with raft spacers, with inset image of the ball nut.

Next, the raft spacers (2.11) need to be placed on top of the heater slide rails. Then, place the linear actuator stage (2.8) on top of them and screw them down to fasten the actuator stage and raft spacers to the heater slide rails. Also, the ball screw connector (2.12) and ball nut (included with ball screw kit) need to be mounted to the bottom of the linear actuator stage (2.8). For reference at this step, see Fig. 6. Note that the fasteners and ball nut are not shown in the bottom left of Fig. 6. The ball nut can be seen in the inset image at the top right of Fig. 6.

**Fig. 7:**
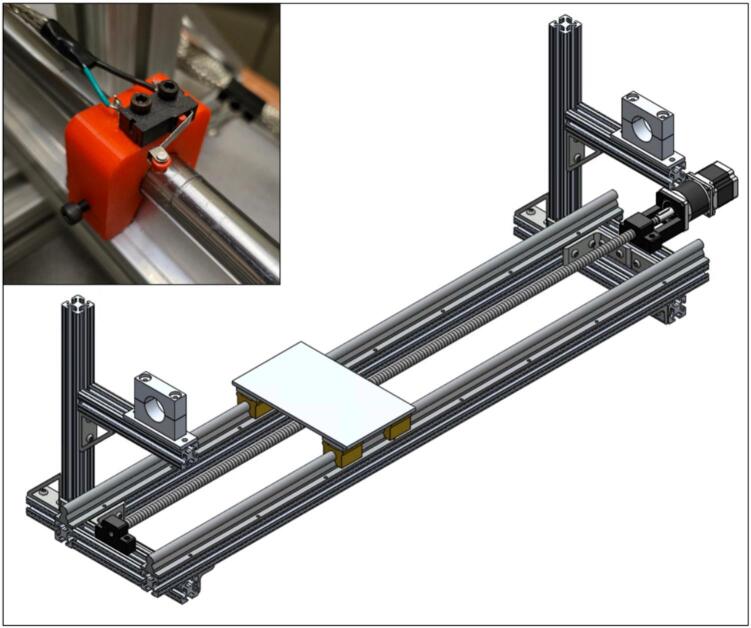
Linear actuator assembly (bottom right), with inset of limit switch mounts (top left).

Next, the NEMA 23 motor and 100:1 gearbox (2.3) need to be mounted to the NEMA 23 ball screw mounting bracket (2.5). Be sure to add the shaft coupling (2.4) to the end of the gearbox. Lastly, install the ball screw (2.1) by feeding it through the screw connector (2.12) and into the ball screw support (2.10). The shaft coupling needs to be tightened to both the gearbox shaft and the ball screw (2.1). See the bottom right of Fig. 7 for reference at this stage. Additionally, the limit switch mounts (2.6) should be fastened to the linear rails closest to the vertical 80/20 bars using a set screw. The limit switches (2.7) can also be mounted at this stage. Please note that the position of the limit switches corresponds to the range of the ring furnace.

### 5.4 Ring furnace

**Fig. 8:**
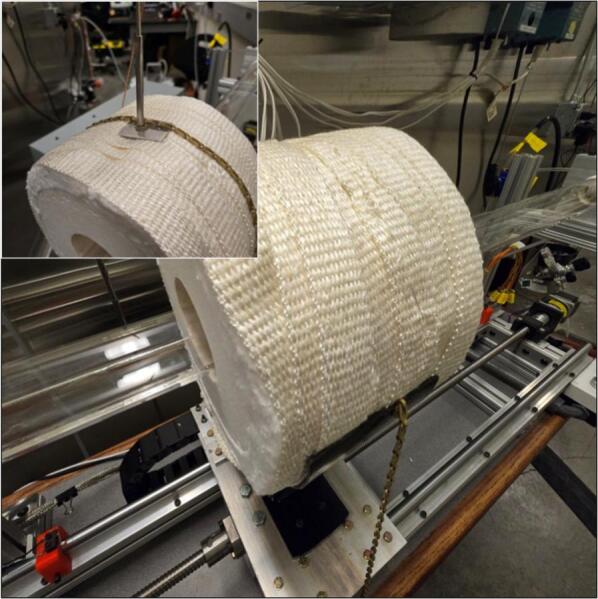
Ring furnace wrapped in ceramic wool insulation and heater insulation wrap (bottom right), with inset image of installed thermocouple mounting rod (top left).

The ring furnace (3.1) needs to be wrapped in ceramic wool insulation (3.2); one wrap around the ring furnace is sufficient. Then cover the ceramic wool insulation (3.2) with the heater insulation wrap (3.6). The specific insulation and wrap are highly recommended as they will not emit fibers when heated. It is recommended to cover the entire outer surface of the furnace with the heater insulation wrap (3.6) to prevent burns during use. Additionally, a hole needs to be drilled through the top of the ring furnace (3.1) and its wraps. A thermocouple mounting steel rod (3.8) should be inserted into this hole to secure the ring furnace thermocouple. For reference, see Fig. 8.

**Fig. 9:**
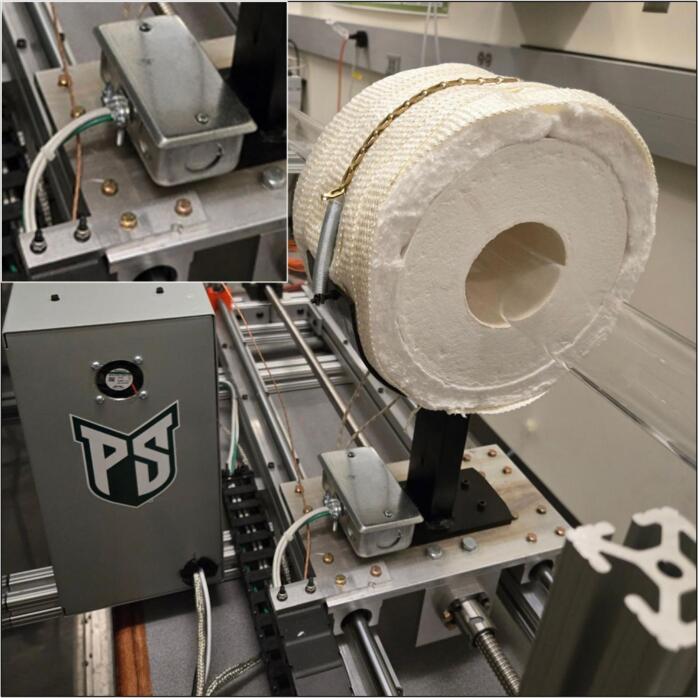
Ring furnace fastened down to heater mount using hold down chain and hold down spring (bottom right), with inset of electrical box on heater mount (top left).

Next, the heater mount (3.5) needs to be fastened to the linear actuator stage (2.8). The heater mount was fabricated out of mild steel. The heater mount (3.5) holds the ring furnace (3.1) high enough to be able to translate over the quartz tube and to be far enough away from any temperature-sensitive components. Two holes should be drilled on either side of the heater mount (3.5). These holes are used to fasten the ring furnace (3.1) down to the heater mount (3.5) using the hold down chain (3.4) and the hold down spring (3.3). Once the heater mount (3.5) is fastened down, the wrapped ring furnace (3.1) can be placed in the heater mount (3.5) and fastened down using the hold down chain (3.4) and hold down spring (3.3). The heater box (3.7) needs to be mounted to the top face of the bottom of the heater mount (3.5). Note that at this stage, wiring components is not recommended; wiring will be covered in Section 5.6. For the final heater assembly, see Fig. 9.

### 5.5 Quartz tube

**Fig. 10:**
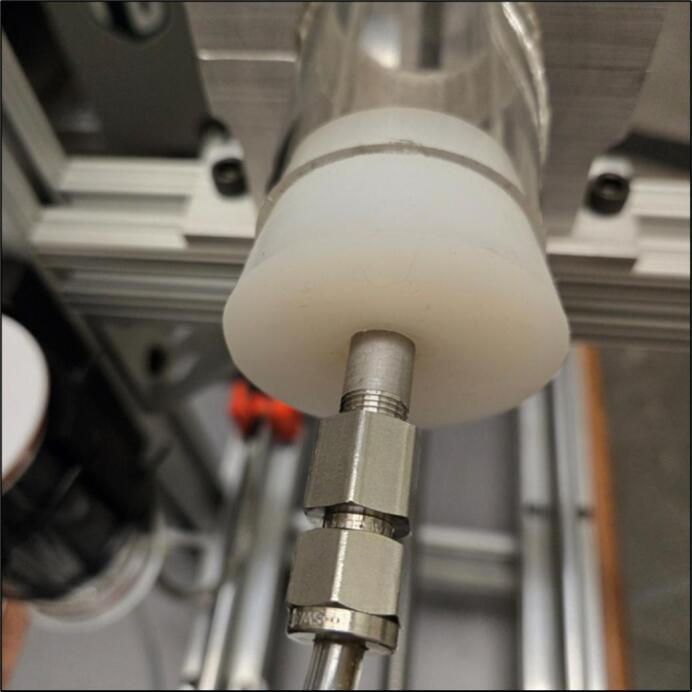
Inlet tube feeding into tapered silicone plug.

To begin the quartz tube assembly, both silicone plugs (4.2) need a hole drilled into them in the center. The diameter of this hole should match the diameter of the tubing being used. In our case, ¼’’ (0.64 cm) swage lock inlet and outlet tubes (4.3) are used. The silicone will provide a seal around the metal tube, preventing any leaks. An example of this assembly is shown in Fig. 10.

**Fig. 11:**
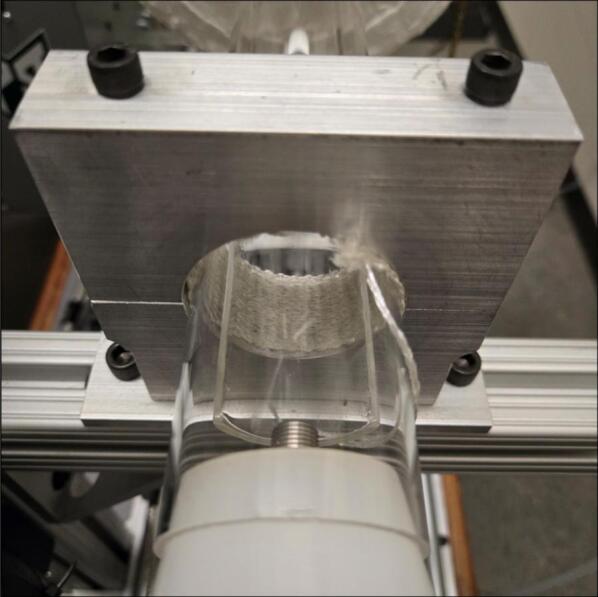
Heat-resistant fabric separating the quartz tube mounts from the quartz tube.

It is also recommended to place temperature-resistant fabric, such as the heater insulation wrap (3.6), for separation of the quartz tube mounts and the quartz tube (4.1). This will reduce the likelihood of the quartz tube mounts being too tight against the quartz tube (4.1). The bolts that hold the quartz tube mounts against each other should only be finger-tight. This can be seen in Fig. 11.

The inlet and outlet tubes (4.3) can be connected to a gas-phase pump of the user’s choice. In our case, a 12 V pump with an upper limit of 3.5 LPM is used.

### 5.6 Electronics

**Fig. 12:**
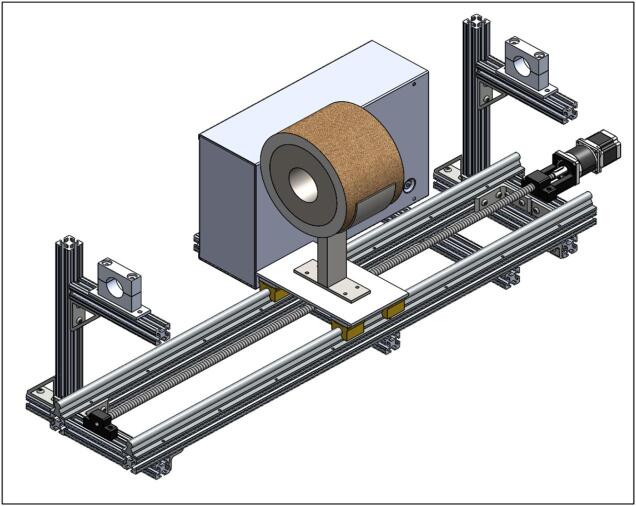
Linear Actuated Quartz Tube Furnace (LA-QTF) with electronics enclosure.

Please note that the quartz tube should be removed from the LA-QTF and stored somewhere safe to prevent damage during the steps in this section. To begin, the electronics enclosure (5.1) should be mounted to the quartz tube furnace if a stand-alone unit is desired, although this is not required. To do this, mount the two 80/20 1515 20'' (50.8 cm) bars (1.3) to the bottom of the frame if you have not done so already. Holes should be drilled through the bottom of the electronics enclosure (5.1) and it should be fastened to the two 80/20 1515 20'' (50.8 cm) bars (1.3) as previously stated. For reference at this step, see Fig. 12.

**Fig. 13:**
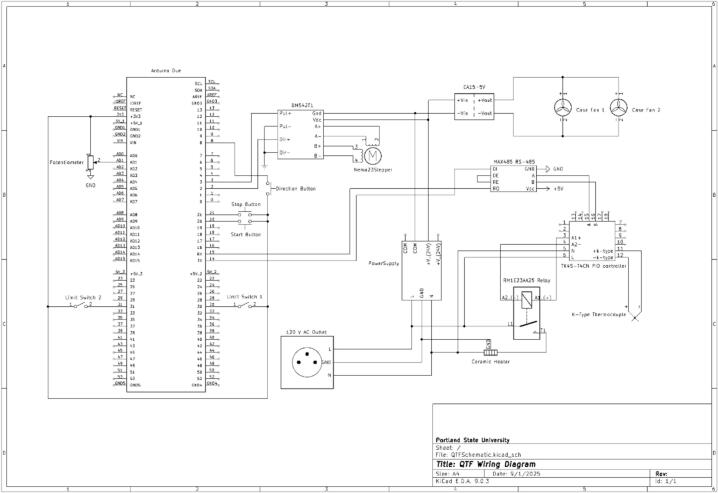
Linear Actuated Quartz Tube Furnace (LA-QTF) wiring diagram.

When following the wiring diagram seen in Fig. 13, test each component as it is installed. It is much easier to troubleshoot each individual component as it is installed rather than at the end; this is general practice for complicated electronic systems. This can be done with a multimeter by testing continuity, voltages, and current values. Another simple way to troubleshoot the system is to run the example code from the manufacturer for each board or sensor and verify it is working as expected; the code can be found on the manufacturer’s website.

**Fig. 14:**
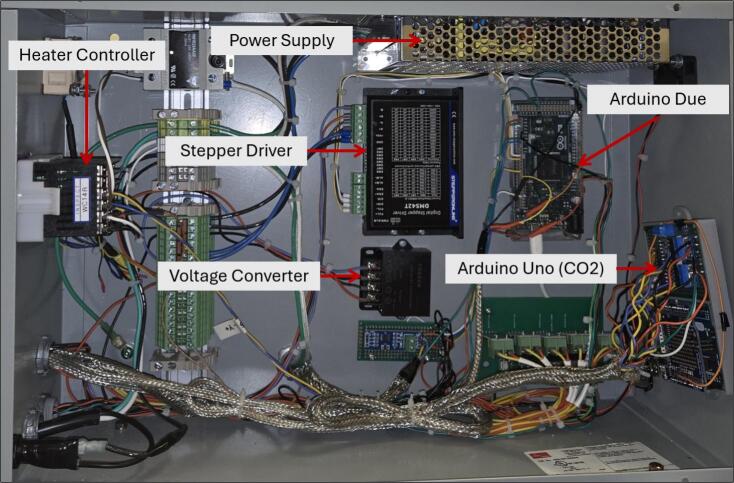
Electronics enclosure internal layout.

We chose to set up our electronics enclosure in the layout seen in Fig. 14; however, the exact positioning of each component is not paramount and may be modified by the user. It is recommended to not run the signal wires near the power wires as electrical interference may occur. This can be further mitigated by wrapping all signal wires in wire shielding.

The electrical system for the LA-QTF consists of four different voltage levels and types, responsible for all of its internal components. The primary power for the device comes from a single wall outlet plug supplying 120 V AC. Notable components that receive power directly from this line are:

• Ceramic heating element (3.1)
• TK4S-14CN – High-performance temperature controller PID (5.3)
• 120 V AC to 24 V DC power supply (5.6)

The next stage uses 24 V DC power from the previous power supply and is responsible for the moving components of the LA-QTF:

• DM542T – Stepper motor driver (5.7)
• NEMA 23 – Stepper motor (2.3)
• 24 V DC to 5 V DC EA15-5 V power converter (5.13)

The 5 V DC power supply is used as an alternative 5 V source to the Arduino Due (5.5), as the Due cannot provide the required current to the necessary components. It also helps isolate the Due from noise generated by these components:

• Two interior 5 V case cooling fans (5.22)
• MAX485 RS-485 range extender (5.8)

The Due itself will be powered by a USB connection to the user’s device of choice. This provides a serial connection where temperature measurements, distance measurements, and debugging can be performed. When plugged in by USB, the Due can provide both 5 V and 3.3 V for low-current components. It should be noted that while the Due can provide a 5 V output, the board itself runs on 3.3 V logic and nothing exceeding that should be connected to the pins directly. The following connections will all use 3.3 V logic:

• Start/stop buttons (5.24)
• Temperature target setting potentiometer (5.27)
• Limit switches (2.7)

Extra care should be taken in isolating these components from the high-power lines serving the ceramic heater and 24 V DC power supply as they are susceptible to noise from these lines, resulting in false positives and potential damage to the Arduino’s input ports. Some form of wire shielding should be used for the signal lines from the limit switches, as those will inevitably run parallel to the heater line.

In terms of general wiring, following the attached circuit diagram will result in a functional LA-QTF device. As components can vary slightly from device to device, we recommend testing individual components prior to incorporation into the overall device. While most components are straightforward for installation and wiring, some are more involved and deserving of further elaboration, which is provided below. Note that not all pins require attachments. Take extra care not to provide incorrect power to any component.

The TK4S-T4CN (5.3) temperature controller has four primary sets of connections. Pins 5 and 6 receive power from the 120 V AC (5.23) supply’s neutral and live lines. Pins 3 and 4 are connected to pins A1 and A2, respectively, on the RM1E23AA25 (5.2) relay. Pins 11 and 12 connect to the K-type thermocouple’s (5.4) negative and positive lines, placed within the ceramic heater to provide temperature control feedback. Pins 15 and 16 connect to pins A and B on the MAX485 (5.8) signal extender, respectively.

The DM542T (5.7) stepper motor controller receives direction (DIR + ) and speed input (PUL + ) from the Due ports 2 and 3, and receives Vdc and GND from the 24 V power supply. A+, A-, B+, B- are all outputs directly to the NEMA 23 stepper motor. Ensure A + and A-, B + and B-, are both paired to their respective stepper motor coils.

The RM1E23AA25 (5.2) solid-state relay is controlled by the TK4S-T4CN’s pins 3 and 4. When activated, it allows current to pass from the main 120 V AC live wire (pin L1) to T1, which is connected to the ceramic heater and returns to the neutral line.

The MAX485 (5.8) signal extender receives 5 Vcc and GND from the Due, A and B from the TK4S-T4CN, and connects its DI and DO ports back to the Due TX and RX ports 14 and 15. DE and RE are shorted together.

All buttons, limit switches, and potentiometers take 3.3 V in from the Due and terminate as inputs on the Due when pressed, activated, or turned.

The following list represents the component hierarchy present in the LA-QTF:

• 120 V AC from wall outlet
  o Ceramic heater (through the RM1E23AA25 relay)
  o TK4S-14CN – High-performance temperature controller PID
  o 120 V AC to 24 V DC power supply
    ▪ DM542T – Stepper motor driver
      • NEMA 23 – Stepper motor
    ▪ 24 V DC to 5 V DC EA15-5V power converter
      • MAX485 RS-485 signal extender
      • Case cooling fans
• 5 V USB power – Arduino Due (3.3 V)
  o Start/stop buttons
  o Temperature target setting potentiometer
  o Limit switches

**Fig. 15:**
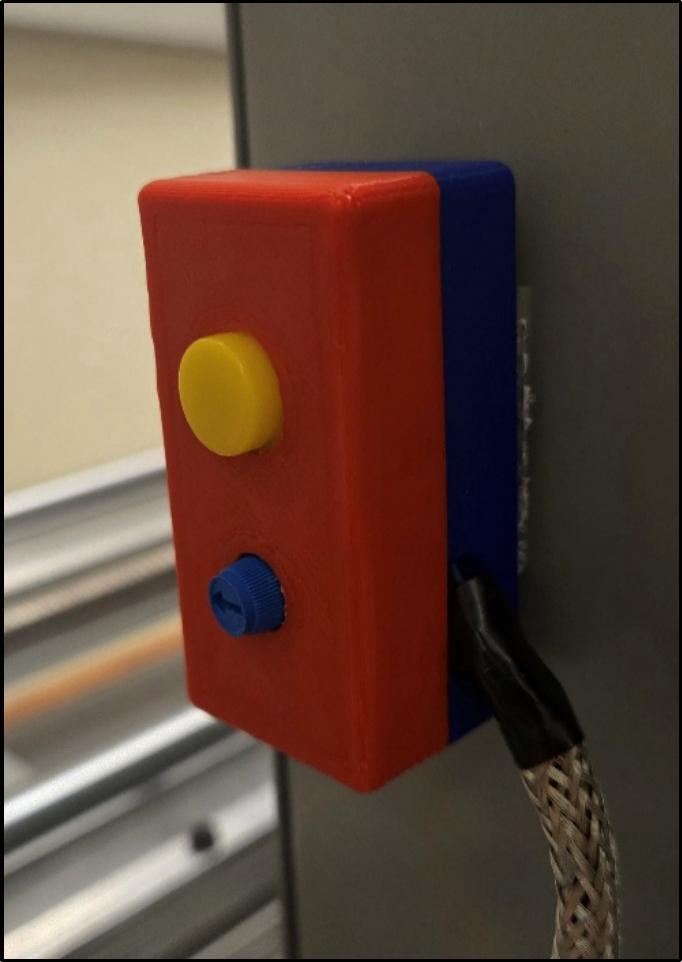
Small enclosure to house temperature and direction control.

Additionally, a small enclosure was designed to house the potentiometer (5.27) that controls temperature and the push button (5.24) that controls motor direction. It is a snap-fit design; the small enclosure top (5.25) snaps into the small enclosure bottom (5.26) without any fasteners, as seen in Fig. 15.

### 5.7 Emissions sensor system (optional)

The SCD-30 CO_2_ monitors are wired to a separate Arduino Uno rather than the Due controller used for other systems. This system could be integrated using the Arduino Due mentioned in the electronics build section, but it proved simpler to have it be a separate electronic system in our case. An I^2^C multiplexer is necessary to use two of the same I^2^C sensors on a single Arduino Uno. Both SCD-30 sensors share the same non-editable addressable ID, making it impossible to call only one without an I^2^C multiplexer. The multiplexer allows you to call individual pins on the multiplexer board, to which the SCD-30 sensors will be connected. Small airtight chambers are used to house CO_2_ sensors to ensure they can sample from inlet and outlet chamber flows, as shown in Fig. 18. Any type of airtight chamber will work if it meets the dimensions in Table 6 and has a removable lid to insert the 3D-printed parts. Note that the emissions sensor system is not represented on the LA-QTF CAD assembly files, though the individual custom 3D-printed part files for mounting the chambers are provided.

**Table 6:** Airtight chamber dimensions for CO_2_ emissions sensor system.

Outer Diameter (in)	Inner Diameter (in)	Inner Height (in)
2.50	2.25	3.00

**Fig. 16:**
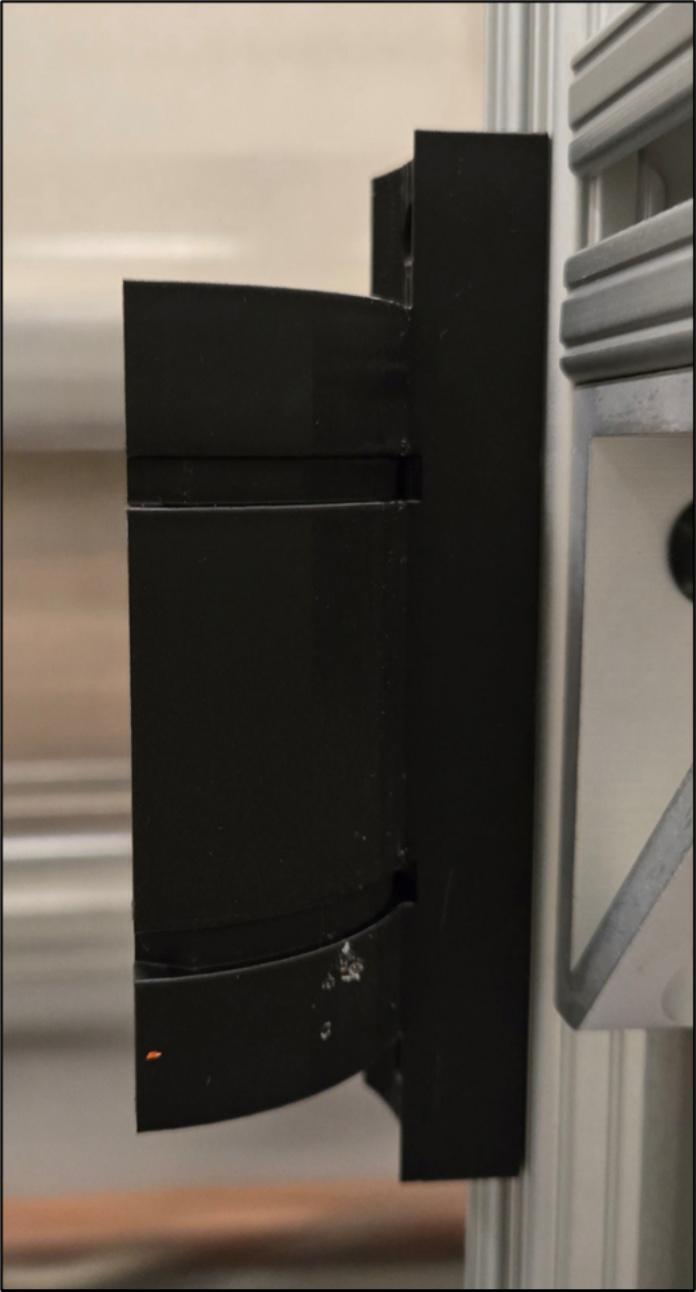
Airtight chamber mounted to vertical 80/20 rail.

The airtight chamber mount (6.3) needs to be fastened to the vertical 80/20 rails, as seen in Fig. 16.

**Fig. 17:**
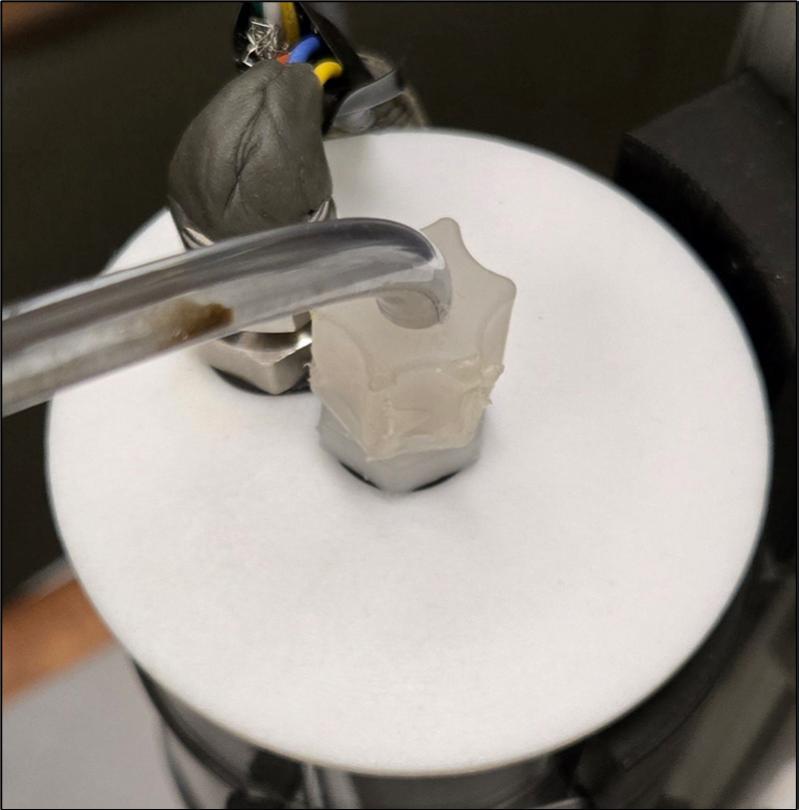
Top of airtight chamber showing putty seal over pierced septum and swage lock fitting.

Next, solder the wires to both SCD-30 sensors (6.1) according to the wiring diagram in Fig. 20. Mount each SCD-30 sensor (6.1) to its own SCD-30 mount (6.6) and insert them into their own airtight chamber. Do not connect the wires to the multiplexer as they still need to be fed through the top of the airtight chamber. In this case, two holes are drilled into the glass chamber top. One is for sample air to pass through using swage lock ¼’’ (0.64 cm) fittings. The other hole is also a swage lock ¼’’ (0.64 cm) fitting, but a pierced septum is placed between the tube and the nut; furthermore, the SCD-30 wires are fed through this pierced septum. The septum acts as a seal around the wires. To ensure a tight seal, putty was added on top of the septum. For reference once assembled, see Fig. 17.

**Fig. 18:**
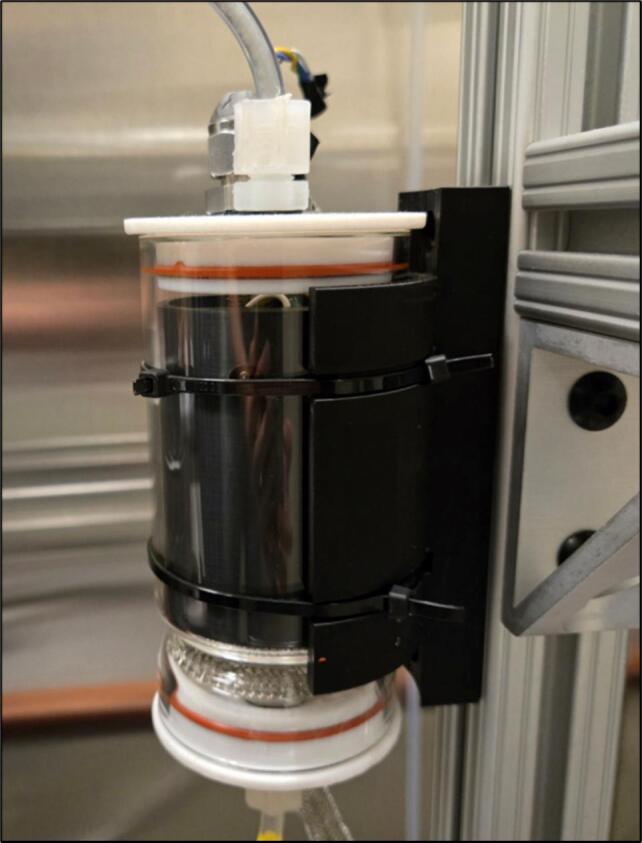
Final assembly of one airtight SCD-30 sensor apparatus.

On the airtight chamber bottom, another hole is drilled to let the air pass through another swage lock ¼’’ (0.64 cm) connection. Next, the airtight chambers (6.4) are mounted to the airtight chamber mounts (6.3) using zip ties, as seen in Fig. 18.

**Fig. 19:**
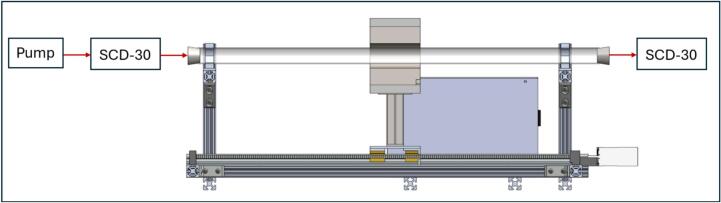
Airflow path for SCD-30 CO_2_ system.

**Fig. 20:**
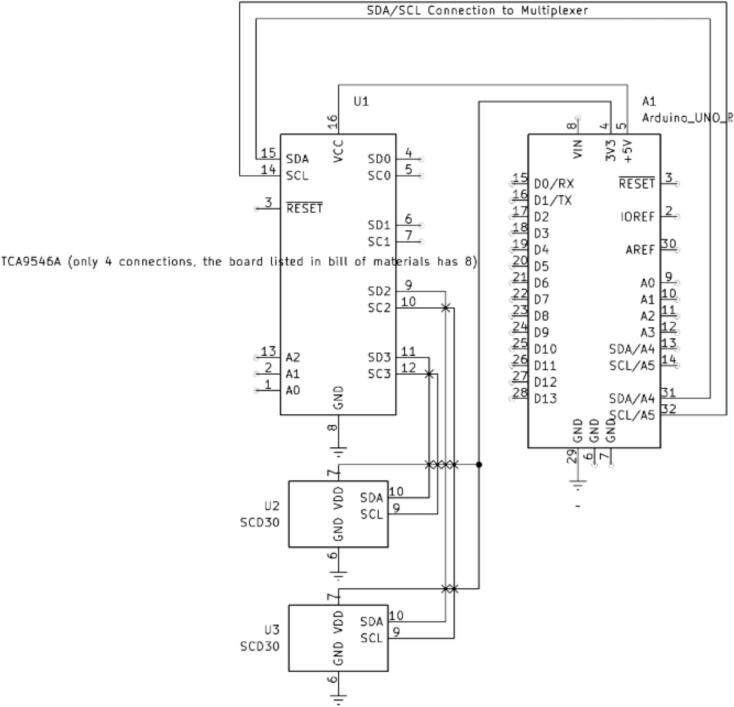
Wiring diagram for SCD-30 or any I^2^C-capable system.

This process is to be repeated on the other side of the quartz tube. Additionally, the airflow path can be seen in Fig. 19, and the wiring diagram for the SCD-30 sensor system is shown in Fig. 20.

To wire the SCD-30 CO_2_ system, follow the wiring diagram in Fig. 20. This diagram is also compatible with any I^2^C sensor by substituting the SCD-30 sensors with the desired alternative.

## 6 Operation instructions

### 6.1 Operation instructions: quartz tube maintenance

To ensure the quartz tube is ready for an experiment, it should be cleaned beforehand with unscented dish detergent. It is important to not contaminate the interior of the tube while using or cleaning it. Gloves should always be worn during the cleaning process or while touching the interior of the quartz tube. After hand-drying the quartz tube, the ring furnace can be run over the tube at 400 °C to dry all remaining moisture. Detailed cleaning instructions are provided below:

1. Thoroughly rinse the quartz tube with tap water.
2. Add unscented dish detergent to the inside of the tube.
3. Thoroughly scrub the interior with a brush.
4. Rinse the tube again.
5. Run a lint-free cloth through the tube to dry the interior to the best of your ability.
6. Rinse the tube once again.
7. Run a new lint-free cloth through the interior of the tube again.
8. Run the furnace over the tube in both directions at 400 °C to eliminate any further contamination.

### 6.2 Operation instructions: experiment preparation

1. Plug the Arduino Due USB into a computer.
2. Plug in the LA-QTF power cable.
3. Open “QTF_9-15–25.ino” via Arduino and select the correct port and Arduino Due (Native USB) under board manager. Upload the code.
4. Press the ‘start motor’ button on the furnace, then the ‘stop motor’ button. This will start and stop the motor. This is necessary to change the temperature setting.
5. Distribute mass evenly in the middle of the glass tube. The furnace should not preheat over the mass or it will combust early.
6. Check that both silicone plugs are plugged into both sides of the quartz tube; otherwise, smoke will leak out of the tube.
7. Plug the pump cable into a wall port.
8. Select the desired temperature via the knob near the yellow button and wait for the system to preheat. Note that the glass tube and the heater will become hot.
9. Plug the Arduino Uno USB into a laptop if CO_2_ data is desired.
10. Open “multiplexer_scd30.ino” via a new Arduino tab and select the correct port and Arduino Uno under board manager. Run the code. Be sure to open the serial monitor on this Arduino tab to record data. To automatically load the Arduino Uno data into an Excel sheet, use the Data Streamer add-in for Excel.

### 6.3 Operation instructions: experimentation

1. Press the ‘start motor’ button to start the stepper motor. Note that the motor will not run if the temperature is set to 0 °C.
2. Wait for the furnace to run over the mass being combusted.
3. Press the ‘stop motor’ button to stop the stepper motor.
4. Press the ‘switch direction’ button to change the direction of the stepper motor.
5. Press the ‘start motor’ button to start the stepper motor and reset the position of the furnace.
6. The limit switch will stop the furnace once it reaches the reset position, but it is a good idea to wait and press the red button to stop it manually.
7. Unplug both USB cables and all power cables.

## 7 Validation and characterization

The key performance metrics presented are 1) temperature characterization at three different setpoints, 2) translation speed, 3) reproducible combustion emission data from experiments conducted using the LA-QTF, and 4) validation of the CO_2_ sensor system alongside the MCE, which indicates consistent combustion conditions.

### 7.1 Quartz tube internal temperature validation

**Fig. 21:**
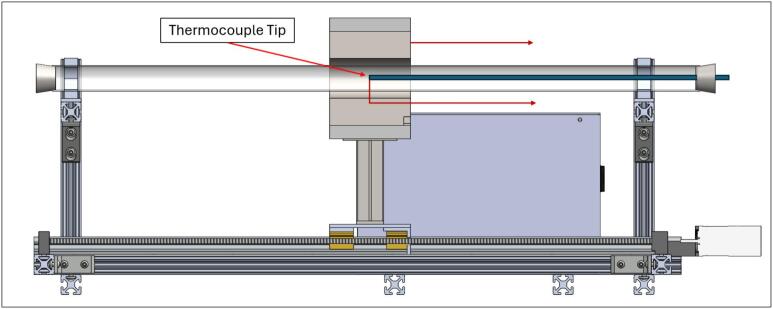
Temperature validation experimental setup.

The ring furnace can heat to 700 °C in the current configuration. Due to heat loss to the environment, convection via airflow through the tube, and the thermal resistance of the quartz tube, the ring furnace temperature is considerably higher than the internal temperature of the quartz tube. It is important to know the internal temperature of the quartz tube, as this is the temperature at which the material will combust. To characterize the true internal temperature of the quartz tube inside the ring furnace, a K-type thermocouple was slowly pulled along its length while the centerline (i.e., half the height of the cylinder) of the ring furnace was translating over the tip of the thermocouple, as seen in Fig. 21.

**Fig. 22:**
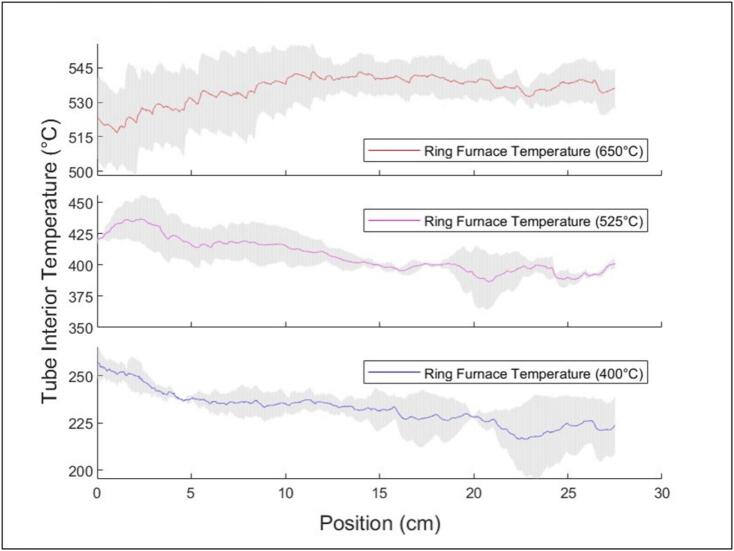
Internal quartz tube temperature at three ring furnace setpoints. The solid color lines represent the average temperature across three replicate tests at each setpoint (400 °C, 525 °C, and 650 °C). The shaded grey regions surrounding each temperature profile represent the standard deviation across replicates.

Shown in Fig. 22 is the measured internal temperature of the quartz tube at three furnace temperature settings (400 °C, 525 °C, and 650 °C), with a pump airflow rate of 3.5 LPM. The ring furnace was translating at a velocity of 1 cm/min. For each temperature, experiments were conducted in triplicate, and the results were averaged. Based on Fig. 22, the LA-QTF is able to hold specific temperature ranges for extended periods of time, though one can observe heat losses resulting in tube temperatures ∼ 100–175 °C lower than furnace setpoint temperatures.

The variability observed in the interior tube temperature, as shown in Fig. 22, is attributed to the preheating process. Prior to reaching the designated operating temperature, the furnace must first warm to the setpoint. During the preheating process, the initial section of the quartz tube experiences unintended heating, resulting in elevated temperatures near the beginning of the tube. Note that the difference between the ring furnace temperature and the measured temperature in the quartz tube is due to losses to the surrounding environment. For the 650 °C data presented in Fig. 22, this is near the maximum temperature of the ring furnace, meaning there will likely be a larger difference between the ring furnace and quartz tube temperatures compared to lower setpoints.

Additional contributors to the variability in the interior tube temperature include convection processes resulting from airflow through the tube and the inherently unpredictable flow patterns within it. Due to the mass transport requirements associated with combustion emission measurements, maintaining a temperature range narrower than ± 25 °C is difficult. For some specific applications, like biomass combustion emission research representing wildfires and WUI fires, this range is acceptable.

### 7.2 Validation of LA-QTF translation speed

**Fig. 23:**
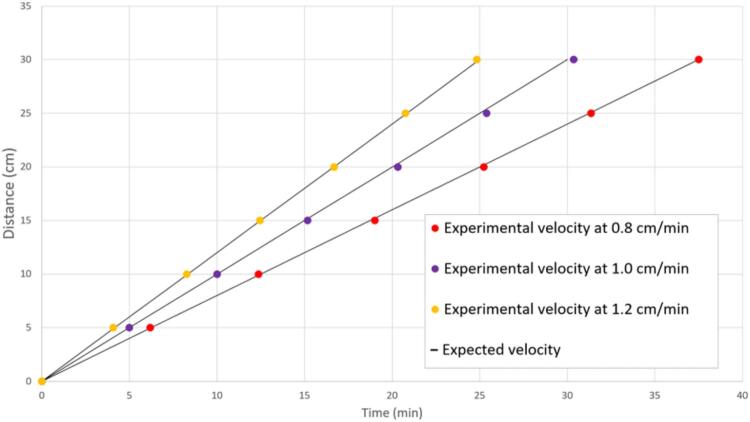
Furnace translation validation at speeds of 0.8 $\frac{\mathrm{cm}}{\min}$, 1.0 $\frac{\mathrm{cm}}{\min}$, and 1.2 $\frac{\mathrm{cm}}{\min}$.

The translation speed of the furnace was validated, as seen in Fig. 23. This speed can be altered within the range of 0.8 $\frac{\mathrm{cm}}{\min}$ to 1.2 $\frac{\mathrm{cm}}{\min}$ by editing the value of the float variable named “heaterSpeed” on line 11 in the QTF_9-15–25.ino file. When deciding on a translation speed, consider the 100:1 gear ratio described in the text.

### 7.3 Emission factor time-series validation

**Fig. 24:**
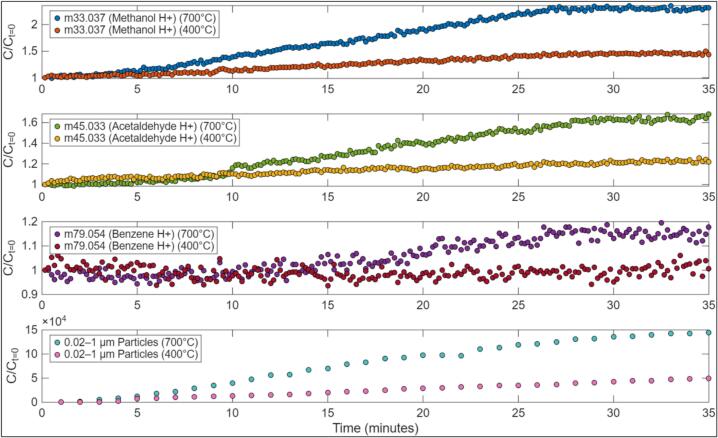
Combustion byproduct generation at two Linear Actuated Quartz Tube Furnace (LA-QTF) ring furnace temperatures: 400 °C and 700 °C.

Prior studies show combustion/heating temperature is a key variable in the generation of pollutants from biomass combustion. For example, aromatic species like polycyclic aromatic hydrocarbons are produced at higher combustion temperatures (>500 °C) [14]. We conducted experiments with the LA-QTF to evaluate the temperature dependence of combustion byproduct generation from heating of dry Douglas fir needles. For two heating conditions (400 °C and 700 °C setpoints on the ring furnace), the flow from the LA-QTF outlet was injected into a large environmental chamber. Concentrations of a variety of combustion byproduct emissions were measured via proton transfer reaction time-of-flight mass spectrometry (PTR-ToF-MS, Ionicon Analytik GmbH, Innsbruck, Austria, PTR1000), while particles were measured with a condensation particle counter (TSI, Model 8525). The large environmental chamber, PTR-ToF-MS, and other measurement tools used to measure concentrations of biomass burning products have been described previously in the literature [15]. Results shown in Fig. 24 demonstrate the consistent generation of combustion byproducts over a ∼ 30-minute period of heating/combustion, with steady increases of methanol, acetaldehyde, benzene, and particles. Results also highlight the effect of heating temperature on pollution generation. For example, lower temperature heating (400 °C setpoint) produces no observable benzene (C/C_t=0_ = ∼1 throughout), while higher temperature heating (700 °C setpoint) shows accumulation of benzene. These results are consistent with prior studies showing elevated production of aromatic organic compounds at higher temperatures.

### 7.4 SCD-30 calibration methods and validation

The SCD-30 sensors were calibrated by injecting CO_2_ into an airtight chamber at multiple known concentrations. A correction factor was developed by plotting the known concentration versus the individual SCD-30 readings, and was implemented into the Arduino code. Eight SCD-30 sensors were calibrated and the two that read closest to one another post-calibration were selected to be placed on the inlet and outlet of the quartz tube.

**Fig. 25:**
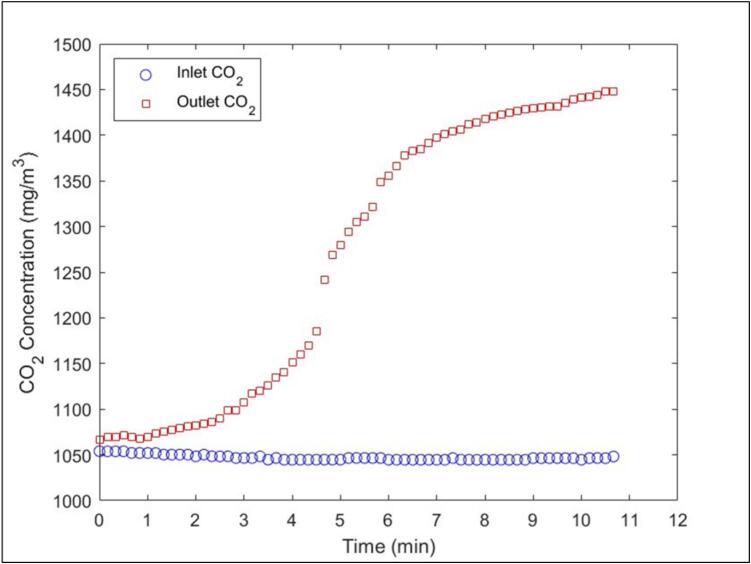
Inlet and outlet CO_2_ concentrations (mg/m^3^) during combustion of 0.2 g of cellulose insulation at a ring furnace temperature of 400 °C.

To validate the SCD-30 CO_2_ sensor system, approximately 0.2 g of cellulose insulation was combusted at a ring furnace temperature of 400 °C. To determine the moisture content of the samples, replicate non-combusted samples were dried in an oven at 80 °C for ∼ 1 h. The ring furnace translated at 1 cm/min over the cellulose insulation alongside an airflow rate of 3.5 LPM. The system was set to take a reading every 10 s. The cellulose insulation was evenly distributed over 10 cm of the quartz tube. The CO_2_ inlet and outlet time-series data of an individual trial can be seen in Fig. 25.

The combustion process increased the CO_2_ concentration by approximately 400 mg/m^3^ over 11 min. CO was measured externally using the Serinus 30 CO monitor in a well-mixed large-scale chamber. The time-series concentrations of both CO and CO_2_ can be converted into emission rates using a discretized mass balance equation specific to our experimental setup. The LA-QTF itself cannot measure MCE and CO emission rates without an external CO monitor. We present these data because they are key performance metrics and strong indicators of combustion regimes. Although not shown here, emission factors can also be calculated [10]. Furthermore, determination of emissions allows for MCE to be calculated Equation (1).

A summary of three experimental results can be seen in Table 7, showing that the LA-QTF can produce repeatable results from replicate trials of a test material at nearly constant moisture content. The uncertainty values reported in Table 7 are the larger of the propagational and experimental uncertainties.

**Table 7:** CO and CO_2_ emission rates and modified combustion efficiency (MCE) from replicate trials during testing of the Linear Actuated Quartz Tube Furnace (LA-QTF).

**Trial**	**Weight (g)**	**Moisture (%)**	**Average CO Emission Rate (mg/h)**	**Average CO_2_ Emission Rate (mg/h)**	**MCE (%)**
1	0.2063	4.392	27	48	64
2	0.1978	3.382	27	37	58
3	0.1960	4.614	23	38	62
AVG	0.2000 ± 0.0002	4.129 ± 0.7	26 ± 2	41 ± 16	61 ± 9

Combustion is an inherently random process. The uncertainty in our quantified emissions presented in Table 7 is consistent with observations of pyrolysis and flaming combustion in other studies [16], which show similar relative uncertainties. For example, across experiments conducted on a variety of biomass and anthropogenic materials, the relative uncertainty of formic acid (a VOC formed in combustion) emissions averaged 62%.

### 7.5 Implications for future studies

The LA-QTF is capable of heating biomass over long-term experiments to create a controlled combustion environment. The device has demonstrated consistent performance, reproducing temperature gradients, expected translation speed, combustion byproducts, CO and CO_2_ emission rates, and modified combustion efficiency values. As human exposure to fire and smoke increases [5], combustion emission research is becoming more relevant. The LA-QTF has the potential to become a widespread laboratory instrument, enabling standardization of small-scale smoldering and flaming combustion emission research. With a shared instrument and repeatable methods across research groups, combustion emission results can be compared and understood. We hope the LA-QTF will support further research into the impacts of WUI fires and other large-scale smoldering or flaming combustion emission events. The construction of this device and its uses are available to all through the open-source license.

## CRediT authorship contribution statement

**Casey Coffland:** Writing – original draft, Visualization, Validation, Software, Investigation, Formal analysis. **Ryan Bixler:** Validation, Software, Investigation. **Elliott Gall:** Writing – review & editing, Supervision, Conceptualization.

## Declaration of competing interest

The authors declare the following financial interests/personal relationships which may be considered as potential competing interests: Elliott Gall reports financial support was provided by United States Environmental Protection Agency. If there are other authors, they declare that they have no known competing financial interests or personal relationships that could have appeared to influence the work reported in this paper.

## Appendix A Supplementary data

Supplementary Data 1 The supplementary data includes a spreadsheet listing the bill of materials for the linear actuated quartz tube furnace build.

## References

[1] Koss A.R., Sekimoto K., Gilman J.B., Selimovic V., Coggon M.M., Zarzana K.J., Yuan B., Lerner B.M., Brown S.S., Jimenez J.L., Krechmer J., Roberts J.M., Warneke C., Yokelson R.J., De Gouw J. (2018). Non-methane organic gas emissions from biomass burning: identification, quantification, and emission factors from PTR-ToF during the FIREX 2016 laboratory experiment. Atmosp. Chem. Phys..

[2] Kocbach Bølling A., Pagels J., Yttri K.E., Barregard L., Sallsten G., Schwarze P.E., Boman C. (2009). Health effects of residential wood smoke particles: the importance of combustion conditions and physicochemical particle properties. Part. Fibre Toxicol..

[3] Lei Y., Lei T.-H., Lu C., Zhang X., Wang F. (2024). Wildfire smoke: health effects, mechanisms, and mitigation. Environ. Sci. Technol..

[4] Bolan S., Sharma S., Mukherjee S., Isaza D.F.G., Rodgers E.M., Zhou P., Hou D., Scordo F., Chandra S., Siddique K.H.M., Bolan N. (2025). Wildfires under changing climate, and their environmental and health impacts. J. Soils Sediments.

[5] S.T. Seydi, J.T. Abatzoglou, M.W. Jones, C.A. Kolden, G. Filippelli, M.D. Hurteau, A. AghaKouchak, C.H. Luce, C. Miao, M. Sadegh, Increasing global human exposure to wildland fires despite declining burned area, (2025). 10.1126/science.adu6408 40839716

[6] Davis A.Y., Cleary T.G., Falkenstein-Smith R.L., Bryant R.A. (2025). Burning characteristics and smoke emission from mixed fuel cribs. ACS EST Air.

[7] Pan X., Kanaya Y., Taketani F., Miyakawa T., Inomata S., Komazaki Y., Tanimoto H., Wang Z., Uno I., Wang Z. (2017). Emission characteristics of refractory black carbon aerosols from fresh biomass burning: a perspective from laboratory experiments. Atmosp. Chem. Phys..

[8] Sun Y., Zhang Q., Li K., Huo Y., Zhang Y. (2022). Trace gas emissions from laboratory combustion of leaves typically consumed in forest fires in Southwest China. Sci. Total Environ..

[9] Garg P., Wang S., Oakes J.M., Bellini C., Gollner M.J. (2024). Variations in gaseous and particulate emissions from flaming and smoldering combustion of Douglas fir and lodgepole pine under different fuel moisture conditions. Combust. Flame.

[10] Kim Y.H., Warren S.H., Krantz Q.T., King C., Jaskot R., Preston W.T., George B.J., Hays M.D., Landis M.S., Higuchi M., DeMarini D.M., Gilmour M.I. (2018). Mutagenicity and lung toxicity of smoldering vs. flaming emissions from various biomass fuels: implications for health effects from wildland fires. Environ. Health Perspect..

[11] Blomqvist P., Hertzberg T., Tuovinen H., Arrhenius K., Rosell L. (2007). Detailed determination of smoke gas contents using a small‐scale controlled equivalence ratio tube furnace method. Fire Mater..

[12] Pokhrel R.P., Gordon J., Fiddler M.N., Bililign S. (2021). Determination of emission factors of pollutants from biomass burning of African fuels in laboratory measurements. J. Geophys. Res. Atmosp..

[13] Trofimova A., Hems R.F., Liu T., Abbatt J.P.D., Schnitzler E.G. (2019). Contribution of charge-transfer complexes to absorptivity of primary brown carbon aerosol. ACS Earth Space Chem..

[14] Laguerre A., Gall E.T. (2024). Polycyclic aromatic hydrocarbons (PAHs) in wildfire smoke accumulate on indoor materials and create postsmoke event exposure pathways. Environ. Sci. Technol..

[15] Stinson B.W., Laguerre A., Gall E.T. (2024). Particle and gas-phase evaluation of air cleaners under indoor wildfire smoke conditions. ACS EST Air.

[16] Dresser W., Ridgway K., Helfrich A., L’Orange C., Jathar S., De Gouw J. (2026). Laboratory analysis of VOC emissions from structural materials in wildland–urban interface fires. Environ. Sci. Technol..

